# Commentary: Phosducin-like 3 is a novel prognostic and onco-immunological biomarker in glioma: a multi-omics analysis with experimental verification

**DOI:** 10.3389/fimmu.2024.1499286

**Published:** 2024-12-18

**Authors:** Jihao Xue, Qijia Yin, Ming Wang, Yanling Li

**Affiliations:** ^1^ Department of Neurosurgery, the Affiliated Hospital, Southwest Medical University, Luzhou, Sichuan, China; ^2^ Department of Urology or Nursing, Dazhou First People’s Hospital, Dazhou, Sichuan, China; ^3^ College of Nursing, Chongqing Medical University, Chongqing, China; ^4^ Department of Rehabilitation or Nursing, the Affiliated Hospital of Southwest Medical University, Luzhou, Sichuan, China

**Keywords:** proportional hazards (PH) assumption, cox regression, global test, glioma, nomogram

We read with great interest the recently reported work by Peng et al., entitled “Phosducin-like 3 is a novel prognostic and onco-immunological biomarker in glioma: A multi-omics analysis with experimental verification” (1). In this study, the authors conducted univariate and multivariate Cox regression analyses in combination with common clinicopathological characteristics and ultimately determined that PDCL3 acted as a potential prognostic biomarker of glioma. They subsequently established a nomogram in the TCGA cohort and confirmed that the nomogram had satisfactory prognostic efficiency for glioma. While we fully recognize the important contributions made by this study, we would like to highlight a few crucial aspects for further scrutiny and analysis. Firstly, it is essential to underscore that the application of Cox regression models necessitates the adherence to the proportional hazards (PH) assumption for the independent variables (2). This means that the hazard ratio associated with each independent variable must remain constant over time (*p* > 0.05). Violation of this assumption can lead to inaccuracies and biases in the statistical inferences drawn from the model (3). In addition, to ensure that the Cox regression model complies with the PH assumption, it is important to conduct a GLOBAL test. A *p*-value greater than 0.05 in this test serves as a strong indicator that the model satisfies the PH assumption, thereby validating its appropriateness for statistical analysis (4).

**Figure 1:**
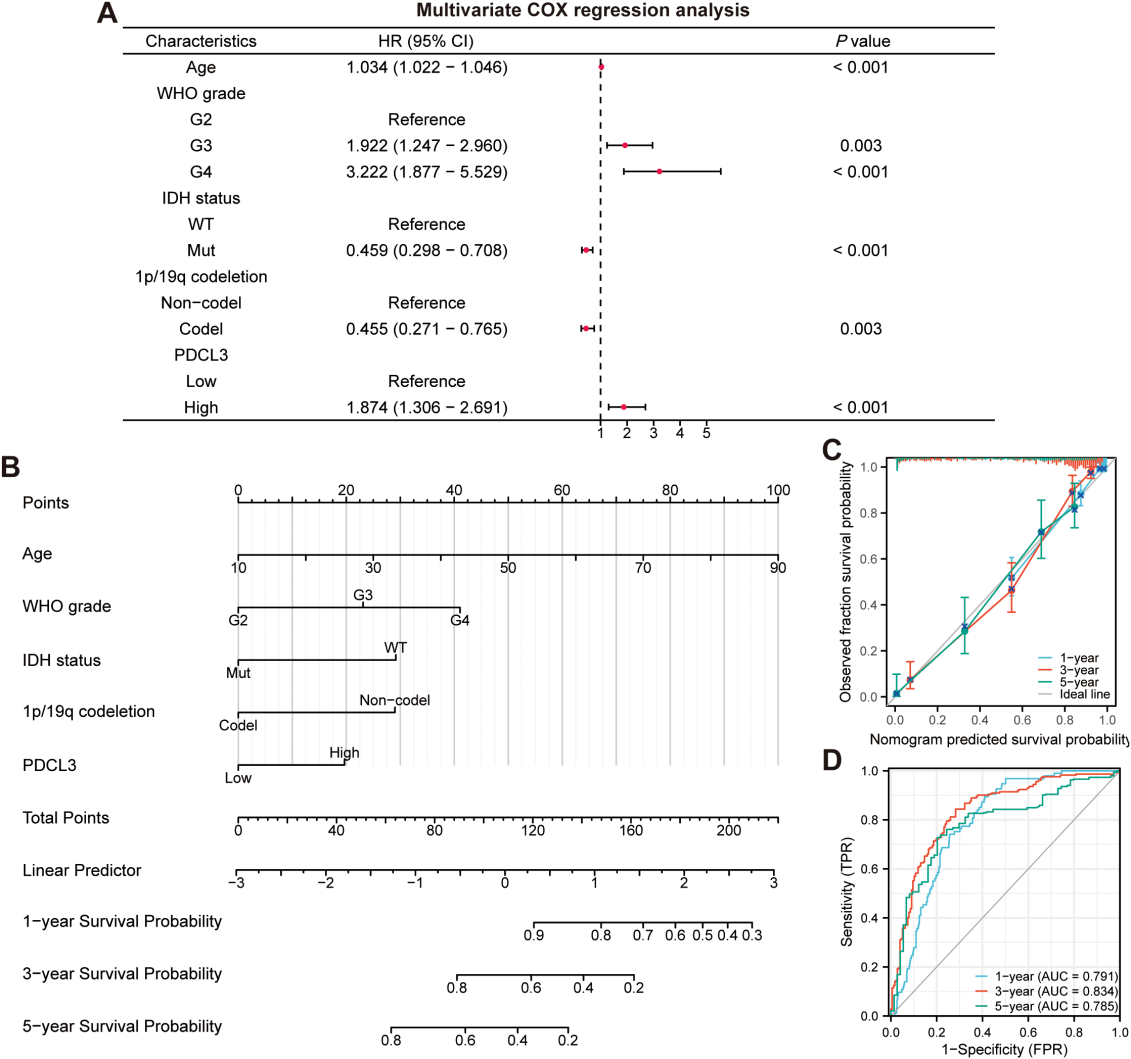
Construction and evaluation of the nomogram. **(A)** Multivariate Cox regression analyses in the TCGA cohort. **(B)** Nomogram based on age, WHO grade, IDH status, 1p/19q codeletion and PDCL3 expression. **(C)** Calibration curves showed the concordance between predicted and observed 1-, 3-, and 5-year OS. **(D)** ROC curve analyses of the nomogram in predicting 1-, 3-, and 5-year OS.

Similarly, we performed univariate and multivariate Cox regression analyses (**Figure 1A**) on the relevant clinical parameters the same as those used by Peng et al. (1) from glioma patients in the TCGA cohort, and tested the PH assumption of multivariate Cox regression (**Table 1**). Subsequently, we constructed a nomogram (**Figure 1B**) based on multivariate Cox regression, and also plotted calibration curves (**Figure 1C**) and ROC curves (**Figure 1D**). The results showed that Age, WHO grade, IDH status, PDCL3 expression levels and GLOBAL did not fulfill the PH hypothesis (**Table 1**), which may affect the credibility and accuracy of the predictive model proposed by Peng et al. (1) from a statistical perspective.

**Table 1:** PH assumption test to multivariate Cox regression.

Variable	Chi-Square Value	Degree of freedom	*P* value
Age	6.426	1	0.011
WHO grade	7.456	2	0.024
IDH status	25.304	1	4.9e-07
1p/19q codeletion	1.465	1	0.226
PDCL3	5.5687	1	0.018
GLOBAL	30.203	6	3.6e-05

If the *p*-value in the GLOBAL test > 0.05, it indicates that the multivariate Cox regression adheres to the PH assumption

In light of the aforementioned considerations, it is imperative to adopt a more rigorous approach to evaluating the predictive capabilities of the model. Firstly, to ensure the robustness and validity of the Cox regression analysis, we propose the inclusion of interaction terms between covariates and time. This refinement enables the model to capture the potential time-varying effects of covariates on the hazard function, thereby addressing the potential violation of the PH assumption (5). Secondly, to further scrutinize the stability of the predictive outcomes, we suggest stratifying the dataset based on the covariates that fail to meet the PH criterion. Within each stratum, separate Cox regression analyses can be conducted, allowing for a more nuanced understanding of the covariate effects within specific subgroups (6). Lastly, we also advocate for the consideration of alternative survival analysis methodologies, particularly accelerated failure time model (7).

Despite our efforts to remind researchers of the prerequisites for using multi-factor Cox regression and constructing nomograms, there are still many papers published that have overlooked the PH assumption in their research. This situation indicates that the rigorous validation of models remains a neglected but crucial aspect in scientific research.
